# Supplementary material for: Parental feeding and childhood genetic risk for obesity: exploring hypothetical interventions with causal inference methods
Source: Int J Obes (Lond). 2022 Mar 19;46(7):1271–9. doi: 10.1038/s41366-022-01106-2 (PMC9239906; doi:10.1038/s41366-022-01106-2)
Supplement: Supplementary file 1 — supplement [file 41366_2022_1106_MOESM1_ESM.docx]

| **Supplementary Information**  **Supplementary Table 1. Items measuring parental feeding practices when children were 10.7 years in ALSPAC** | | | | | | | |
| --- | --- | --- | --- | --- | --- | --- | --- |
|  | **Response options, N(%)** | | | | |  |  |
| **Items** | **Disagree** | **Slightly disagree** | **Neither agree or disagree** | **Slightly agree** | **Agree** | **Factor loadings** | **Subscale derived from factor analysis** |
| I have to be sure that she does not eat too many sweets | 792 (11) | 296 (4) | 886 (12) | 2237 (30) | 3129 (43) | 0.53 | Restriction |
| I have to be sure that she does not eat too many of her favourite foods | 2103 (29) | 806 (11) | 2096 (29) | 1520 (21) | 788 (11) | 0.71 | Restriction |
| I deliberately keep some foods out of her reach | 4772 (65) | 414 (6) | 724 (10) | 748 (10) | 652 (9) | 0.67 | Restriction |
| It’s OK to offer sweets as a reward for good behaviour | 1875 (26) | 945 (13) | 1738 (24) | 1741 (24) | 1030 (14) |  | Did not load on specific factor, and hence removed |
| If I did not guide or regulate her eating she would eat too much | 4369 (60) | 568 (8) | 686 (9) | 1025 (14) | 670 (9) | 0.7 | Restriction |
|  | **Never** | | **Sometimes** | | **Always** |  |  |
| I insist that she eats all the food on the plate | 2847 (41) | | 3606 (51) | | 572 (8) | 0.75 | Pressure to eat |
| If she does not finish all of the main course she is not allowed a pudding | 2487 (37) | | 3401 (51) | | 792 (12) | 0.73 | Pressure to eat |
| I tell her off for playing or fiddling with food at mealtimes | 2481 (39) | | 3347 (53) | | 538 (8) | 0.53 | Pressure to eat |
| I allow her to eat only at meal times, and not in between meals | 3200 (47) | | 3408 (50) | | 187 (3) |  | Did not load on specific factor, and hence removed |
| I cheer her up with something to eat if she is sad or upset | 3384 (50) | | 3339 (49) | | 79 (1) | 0.56 | Emotional feeding |
| I like to take her out for a special meal when something good happens to her | 1573 (23) | | 5017 (73) | | 317 (5) | 0.67 | Emotional feeding |
| I give her her favourite food when she is hurt or sick | 1414 (21) | | 4748 (69) | | 736 (11) | 0.71 | Emotional feeding |
| I like to prepare a special meal for her when something good happens to her | 1434 (21) |  | 5019 (73) |  | 462 (7) | 0.82 | Emotional feeding |

| **Supplement Table 2**. Means and standard deviations of standardized parental feeding behaviors and Body Mass Index at 12 years across the 5 quintiles of the PGS-BMI, n= 4,248 | | | | | |
| --- | --- | --- | --- | --- | --- |
|  | **PGS-BMI** | **Restriction** | **Emotional Feeding** | **Pressure to eat** | **BMI at 12 years** |
|  | Mean (SD) | Mean (SD) | Mean (SD) | Mean (SD) | Mean (SD) |
| **1^st^ quintile**  **Lowest risk (N=849)** | -1.46 (0.49) | -0.13 (0.95) | -0.04 (0.97) | 0.03 (0.99) | 17.35 (2.36) |
| **2^nd^ quintile**  **Lower risk (N=850)** | -0.58 (0.17) | -0.09 (1.01) | -0.01 (1.01) | -0.01 (0.97) | 18.13 (2.67) |
| **3^rd^ quintile**  **Average risk (N=849)** | -0.04 (0.14) | -0.06 (0.97) | 0.02 (0.98) | -0.04 (1.02) | 18.96 (3.20) |
| **4^th^ quintile**  **Higher risk (N=850)** | 0.49 (0.16) | 0.07 (0.99) | 0.03 (1.02) | 0.04 (1.00) | 19.53 (3.32) |
| **5^th^ quintile**  **Highest risk (N=850)** | 1.33 (0.43) | 0.2 (1.04) | 0.00 (1.02) | -0.01 (1.01) | 20.64 (3.61) |

Abbreviations: PGS-BMI = Polygenic Score BMI

| **Supplementary Table 3**. Pairwise Pearson’s correlations between exposures, mediators, and outcome; n=4,248 | | | | | |
| --- | --- | --- | --- | --- | --- |
|  | **PGS-BMI** | **Restriction** | **Emotional feeding** | **Pressure to eat** | **BMI at 12 years** |
| **PGS-BMI** | 1 |  |  |  |  |
| **Restriction** | 0.12 | 1 |  |  |  |
| **Emotional feeding** | 0.02 | 0.11 | 1 |  |  |
| **Pressure to eat** | <0.001 | -0.23 | -0.22 | 1 |  |
| **BMI at 12** | 0.36 | 0.3 | <0.001 | 0.1 | 1 |

Abbreviations: PGS-BMI = Polygenic Score BMI; BMI= Body Mass Index
